# Saccharated ferric oxide attenuates haematopoietic response induced by epoetin beta pegol in patients undergoing haemodialysis

**DOI:** 10.1186/s12882-021-02320-2

**Published:** 2021-04-08

**Authors:** Takahide Iwasaki, Akira Fujimori, Takeshi Nakanishi, Shioko Okada, Nobuto Hanawa, Yukiko Hasuike, Takahiro Kuragano

**Affiliations:** 1 Internal Medicine (Nephrology and Dialysis), Hyogo College of Medicine, Mukogawa-cho, Nishinomiya City, Hyogo 663-8501 Japan; 2 Department of Nephrology, Konan Medical Centre, 1-5-16 Kamokogahara, Higashinada-ku, Kobe, 658-0064 Japan; 3 Department of Internal Medicine (Nephrology), Sumiyoshigawa Hospital, 5-6-7 Konan-cho, Higashinada-ku, Kobe, 658-0084 Japan

**Keywords:** Fibroblast growth factor 23, Erythroferrone, Hepcidin, Epoetin beta pegol, Saccharated ferric oxide

## Abstract

**Background:**

Decreased erythropoietin levels and impaired iron metabolism due to excessive hepcidin levels are responsible for renal anaemia in patients undergoing haemodialysis. Recently, erythroferrone (ERFE) has been identified as a factor that regulates hepcidin. In addition, fibroblast growth factor 23 (FGF23), which has been recognized as a phosphorus-regulating hormone, appears to be involved in haematopoietic regulation. Clarification of the detailed mechanism of haematopoiesis could lead to the improvement of renal anaemia treatment.

**Methods:**

Epoetin beta pegol (CERA) was administered to patients undergoing haemodialysis at week 0, and the same amount of CERA with saccharated ferric oxide (SFO) was administered at week 4. The changes in haematopoiesis-related biomarkers, including ERFE, intact FGF23 (iFGF23), C-terminal FGF23 (cFGF23), and inflammatory markers, were examined.

**Results:**

Administration of CERA increased ERFE levels, decreased hepcidin levels, and stimulated iron usage for haematopoiesis, leading to an increase in reticulocytes (Ret) and haemoglobin (Hb). Simultaneous administration of SFO with CERA (CERA + SFO) significantly attenuated the responses of ERFE, Ret, and Hb compared with CERA alone. Although iFGF23 levels were not affected by either CERA or CERA + SFO, cFGF23 was significantly elevated from baseline after CERA. Since cFGF23 levels were not affected by CERA + SFO, cFGF23 levels after CERA + SFO were significantly lower than those after CERA alone. The ratio of iFGF23 to cFGF23 (i/cFGF23 ratio) was significantly higher after CERA + SFO than that after CERA alone. In addition, high-sensitivity C-reactive protein (hsCRP) levels were significantly higher after CERA + SFO than after CERA alone.

**Conclusion:**

Administration of SFO suppressed haematopoietic responses induced by CERA. Elevation of i/cFGF23 ratio and hsCRP could account for the inhibitory effects of SFO on haematopoiesis.

**Trial registration:**

This study was registered with the University Hospital Medical Information Network (ID UMIN000016552).

**Supplementary Information:**

The online version contains supplementary material available at 10.1186/s12882-021-02320-2.

## Background

Absolute iron deficiency, functional iron deficiency, and/or a combination of both are the major obstacles for efficient therapy of renal anaemia with erythropoiesis-stimulating agents (ESAs). Therefore, iron administration is believed to accelerate erythropoiesis. However, as we recently reviewed, excessive iron storage may even impede erythropoiesis or the differentiation of erythroid precursors in the therapy for renal anaemia in patients undergoing haemodialysis [1]. We hypothesized that a major cause of the unresponsiveness to ESA in patients with massive iron storage could be an increase in hepcidin levels.

Hepcidin has been established as a master coordinator of iron metabolism, and its role and regulation have been extensively studied over the past two decades [2, 3]. Recently, an increase in hepcidin level has been presumed to be one of the major factors of renal anaemia, because animal models of chronic kidney disease (CKD), as well as sepsis, did not present anaemia or iron deficiency when hepcidin was knocked out [4, 5]. Focusing on the major regulators of hepcidin, the expression level of hepcidin is upregulated by the iron load and inflammation, and downregulated by erythropoiesis and hypoxia [1, 3]. Regarding erythropoiesis, the erythroid hormone, erythroferrone (ERFE), has been proposed as the potential link between erythropoiesis and iron homeostasis [6, 7]. In response to the stimulation of erythropoiesis by ESA, ERFE that is secreted by erythroblasts in the bone marrow acts on hepatocytes to suppress hepcidin [7, 8]. Iron administration raises hepcidin, but co-administration of ESA may suppress hepcidin elevation via ERFE. It is necessary to investigate the reaction of hepcidin to ESA and iron administration, and the relationship between hepcidin and haematopoiesis in the clinical setting.

In patients with CKD, an increase in fibroblast growth factor 23 (FGF23), which is a bone-derived hormone essential for the homeostasis of mineral metabolism, is observed. First, FGF23 secretion has been demonstrated to be triggered by an increase in phosphorus load from the early stage of CKD. The current findings show that it is also induced by iron deficiency, ESA administration, inflammation, hypoxia, etc. [9, 10]. FGF23 can provoke cardiotoxicity via left ventricular hypertrophy [11], immunodeficiency via suppression of neutrophil migration [12], and inflammation via interleukin 6 (IL-6) production [13]. Recent observations strongly suggest that FGF23 metabolism may also be associated with renal anaemia and responsiveness to ESA. FGF23 knockout mice became polycythemic through decreased apoptosis of erythroid progenitor cells and increased erythropoietin (EPO), whereas administration of FGF23 decreased endogenous EPO and exacerbated anaemia [14]. Commercially available enzyme-linked immunosorbent assays (ELISAs) can quantify the concentrations of intact FGF23 (iFGF23) and C-terminal FGF23 fragments (cFGF23). The source of cFGF23 fragments is FGF23 transcription and subsequent peptide cleavage in the bone. Iron deficiency, inflammation, and EPO have been reported to accelerate FGF23 cleavage [15, 16]. In animal studies, simultaneous measurement of iFGF23 and cFGF23 showed that cFGF23 could attenuate the inhibitory action of iFGF23 on erythropoiesis and iron metabolism [17, 18]. If iron administration suppresses cleavage of iFGF23 and increases the ratio of iFGF23 to cFGF23 (i/cFGF23 ratio), simultaneous replenishment of iron could attenuate haematopoiesis induced by ESA.

The effect of intravenous iron on haematopoiesis, when administered simultaneously with ESA in the treatment of renal anaemia, has not been clarified. In the present study, we tested the effect of co-administration of intravenous iron with ESA on iron and haematopoiesis-related biomarkers, including hepcidin and ERFE, in patients on maintenance haemodialysis. The effects on FGF23 metabolism and inflammatory markers were also studied to investigate the possibility that iron adversely affects haematopoiesis through these factors including hepcidin.

## Methods

### Study design

The study was conducted in Konan Medical Center in March 2015. Patients included in the study were those on haemodialysis treated in Konan Medical Center for more than 12 months with haemoglobin (Hb) levels controlled from 9.5 to 12.0 g/dL with epoetin beta pegol (CERA). The CERA dose had been adjusted according to the renal anaemia guidelines of the Japanese Society for Dialysis Therapy [19]. Exclusion criteria were (i) age under 18 or over 90 years old; (ii) active infectious disease or malignant disease; (iii) uncontrolled hypertension; (iv) iron replenishment within 3 months; (v) serum ferritin > 300 ng/mL. This study was at first planned to be performed for 50 patients. However, we decided to reduce the number of patients to measure more biomarkers and conducted this study as a pilot study. The effects of CERA alone and CERA simultaneously with saccharated ferric oxide (SFO; CERA + SFO) were observed for 4 weeks and compared with each other. After adjusting the dose according to the guidelines, 119 ± 39 μg of CERA (Mircera™, Chugai Pharmaceutical Co. Ltd., Tokyo, Japan) was administered at week 0. The same amount of CERA together with 40 mg of SFO (Fesin™, Nichi-Iko Pharmaceutical, Toyama City, Japan) was administered at week 4. Weekly monitoring of ERFE, hepcidin-25 (HEPC), ferritin (FRN), transferrin saturation (TSAT), reticulocytes (Ret), reticulocyte haemoglobin content (CHr), Hb, iFGF23, cFGF23, high-sensitivity C-reactive protein (hsCRP), and IL-6 levels was carried out for 8 weeks.

HEPC was measured by liquid chromatography-tandem mass spectrometry (LC/MS/MS, Medical Care Proteomics Biotechnology Co. Ltd., Ishikawa Prefecture, Japan) as previously reported [20]. Serum ERFE levels were determined using an ELISA kit (Intrinsic LifeSciences, La Jolla, CA, USA) based on a well-validated method by Ganz et al. [21]. Serum iFGF23 (pg/mL) and cFGF23 (pmol/L) levels were measured using sandwich ELISA kits (Kainos Laboratories, Tokyo, Japan, and Biomedica Medizinprodukte GmbH&CoKG, Vienna, Austria, respectively). The unit conversion of cFGF23 was performed using the following equation: 0.133 pmol/L = 1 pg/mL (molecular weight 7.52 kDa). i/cFGF23 ratio was calculated after the units were unified as pg/mL. IL-6 levels were measured using a sandwich ELISA kit (Proteintech Group Inc., IL, USA) and hsCRP levels were measured using an ELISA kit (Hycult Biotech Inc., PA, USA).

### Ethical considerations

Written informed consent to participate and for publication was obtained from all patients. The study protocol was approved by the Ethics Committee of our hospital (approval number 26–03), and the study was carried out in accordance with the principles outlined in the Declaration of Helsinki. This study was registered with the University Hospital Medical Information Network (ID UMIN000016552).

### Statistical analysis

Data are expressed as mean ± standard deviation or median (interquartile ranges) with *p* < 0.05 considered statistically significant. Data are shown with weeks 0 and 4 as baselines to compare the effects of CERA alone and CERA + SFO. Changes are expressed as the differences from the baseline for ERFE, HEPC, FRT, TSAT, Ret, CHr, Hb, hsCRP, and IL-6 levels. Ratios to the baseline are shown for the changes in iFGF23 and cFGF23 levels. Changes in i/c FGF23 ratios are shown with the calculated values as they are. Data were statistically analysed using a repeated measures ANOVA test. Multiple (and pairwise) comparisons were performed using a Bonferroni post hoc test, using BellCurve for Excel (Social Survey Research Information Co. Ltd., Tokyo, Japan). Pearson’s correlation coefficient was used to determine the correlation between the two variables.

## Results

### Baseline characteristics of the patients

Table 1 shows the patients’ baseline characteristics. There were 4 men and 5 women with an average age of 73.6 ± 6.2 years. The median (interquartile range) of dialysis vintage was 11 (7–19) years. The primary diseases were diabetic nephropathy, chronic glomerulonephritis, and polycystic kidney disease in 3, 5, and 1 patient, respectively. The average Hb level was 10.4 ± 0.6 g/dL, TSAT 38.4 ± 11.9%, FRN 4.9 ± 34.5 ng/mL, albumin (Alb) 3.5 ± 0.2 g/dL, corrected calcium (CCa: calcium + 4 - Alb, if Alb was below 4) 8.8 ± 0.4 mg/dL, inorganic phosphorus (IP) 5.1 ± 0.8 mg/dL. The median (interquartile range) of parathyroid hormone 1–84 (Whole PTH) was 94.6 (68.9–150) pg/mL.

**Table 1:** Patients’ baseline characteristics

Male/female	4/5
Age(y)	73.6 ± 6.2
Dialysis vintage(y)	11 (7–19)
Primary disease	DM:3/CGN:5/PCK:1
Hb(g/dL)	10.4 ± 0.6
Ret (10^4^/mL)	2.1 ± 0.8
TSAT(%)	38.4 ± 11.9
CHr (pg)	33.6 ± 1.0
FRN (ng/mL)	98.9 (63.4–122.7)
HEPC (ng/mL)	0.5 ± 0.3
ERFE (ng/mL)	1.4 ± 0.8
iFGF23 (pg/mL)	665 (164–879)
cFGF23 (pg/mL)	115 (62–198)
hsCRP (ng/dL)	0.11 (0.06–1.05)
IL-6 (pg/dL)	17.2 ± 22.9
Alb(g/dL)	3.5 ± 0.2
CCa (mg/dL)	8.8 ± 0.4
IP (mg/dL)	5.1 ± 0.8
Whole PTH (pg/dL)	94.6 (68.9–150)
CERA dosage at weeks 0 and 4(μg/4 W)	109 ± 39

Mean ± SD or median (interquartile ranges) are shown for continuous variables

*Abbreviations*: *y* years, *DM* diabetes mellitus, *CGN* chronic glomerulonephritis, *PCK* polycystic kidney, *Hb* hemoglobin, *Ret* reticulocyte, *TSAT* transferrin saturation, *CHr* reticulocyte hemoglobin content, *FRN* ferritin, *HEPC* hepcidin-25, *ERFE* erythroferrone, *iFGF23* intact fibroblast growth factor 23, *cFGF 23* C-terminal fibroblast growth factor 23, *hsCRP* high-sensitivity C-reactive protein, *IL-6* interleukin-6, *Alb* albumin, *CCa* corrected calcium, *IP* inorganic phosphorus, *Whole PTH* parathyroid hormone 1–84, *CERA* epoetin beta pegol, *w* week

### Changes in the haematopoietic markers over time

**Fig. 1:**
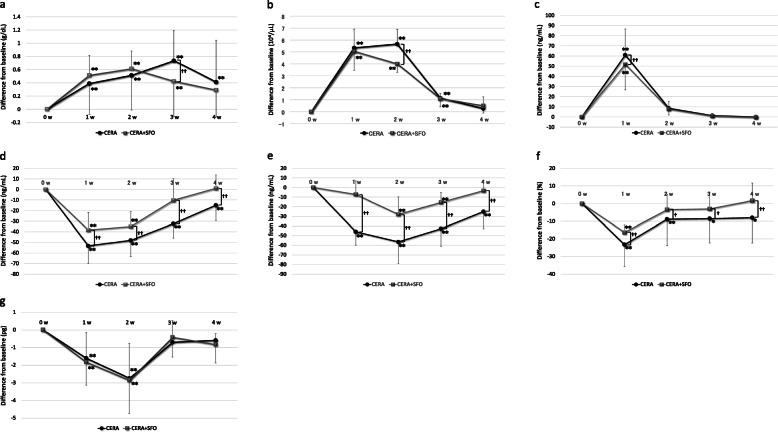
**a**. Temporal changes in Hb from baseline. Means ± standard deviations of the differences from baseline are shown. ^**††**^*p* < 0.01, **p < 0.01 vs. baseline. Abbreviations: Hb haemoglobin, CERA epoetin beta pegol, SFO saccharated ferric oxide, w week. **b**. Temporal changes in Ret from baseline. Means ± standard deviations of the differences from baseline are shown. ^**††**^*p* < 0.01, **p < 0.01 vs. baseline. Abbreviations: Ret reticulocyte, CERA epoetin beta pegol, SFO saccharated ferric oxide, w week. **c**. Temporal changes in ERFE from baseline. Means ± standard deviations of the differences from baseline are shown. ^**††**^*p* < 0.01, **p < 0.01 vs. baseline. Abbreviations: ERFE erythroferrone, CERA epoetin beta pegol, SFO saccharated ferric oxide, w week. **d**. Temporal changes in HEPC from baseline. Means ± standard deviations of the differences from baseline are shown. ^**††**^*p* < 0.01, **p < 0.01 vs. baseline. Abbreviations: FRN ferritin, CERA epoetin beta pegol, SFO saccharated ferric oxide, w week. **e**. Temporal changes in FRN from baseline. Means ± standard deviations of the differences from baseline are shown. ^**††**^*p* < 0.01, **p < 0.01 vs. baseline. Abbreviations: HEPC hepcidin-25, CERA epoetin beta pegol, SFO saccharated ferric oxide, w week. **f**. Temporal changes in TSAT from baseline. Means ± standard deviations of the differences from baseline are shown. ^**†**^*p* < 0.05, ^**††**^*p* < 0.01, *p < 0.05 vs. baseline, **p < 0.01 vs. baseline. Abbreviations: TSAT transferrin saturation, CERA epoetin beta pegol, SFO saccharated ferric oxide, w week. **g**. Temporal changes in CHr from baseline. Means ± standard deviations of the differences from baseline are shown. **p < 0.01 vs. baseline. Abbreviations: CHr reticulocyte haemoglobin content, CERA epoetin beta pegol, SFO saccharated ferric oxide, w week

Figure 1a–g shows a comparison of the temporal changes in the haematopoietic markers after administration of CERA alone and CERA + SFO. Hb levels significantly increased from baseline at weeks 1 to 4 after the administration of CERA (Fig. 1a). After CERA + SFO, significant increases in Hb were not observed at week 4, and a significantly lower level of Hb compared with CERA alone was noted at week 3. Rets significantly increased from baseline after administration of both CERA and CERA + SFO at weeks 1 to 3 (Fig. 1b). Ret levels at week 2 were significantly lower after CERA + SFO than after CERA alone. ERFE showed a significant increase from baseline after administration of both CERA and CERA + SFO only at week 1 (Fig. 1c). A significantly lower level of ERFE was observed with CERA + SFO administration than with CERA alone at week 1. Although the values fluctuated with drug administration, Rets and ERFE showed a very strong correlation (Supplementary Figure S1). HEPC showed significant decreases from baseline at weeks 1 to 4 after CERA administration and weeks 1 and 2 after CERA + SFO administration (Fig. 1d). Administration of CERA alone induced significantly lower levels of HEPC than with CERA + SFO at weeks 1 to 4. ERFE and HEPC showed a strong negative correlation (Supplementary Figure S2). FRT showed significant decreases from baseline at weeks 1 to 4 after administration of CERA and at weeks 2 and 3 after administration of CERA + SFO (Fig. 1e). Administration of CERA alone caused significantly lower levels of FRN compared with CERA + SFO at weeks 1 to 4. TSAT showed significant decreases from baseline at weeks 1 to 4 after administration of CERA and only at week 1 after administration of CERA + SFO (Fig. 1f). Administration of CERA alone caused significantly lower levels of TSAT compared with CERA + SFO at weeks 1 to 4. CHr showed significant decreases from baseline at weeks 1 and 2 after administration of both CERA and CERA + SFO (Fig. 1g). Significant differences were not found in the CHr levels between administration of CERA and CERA + SFO.

### Changes in FGF23 levels over time

**Fig. 2:**
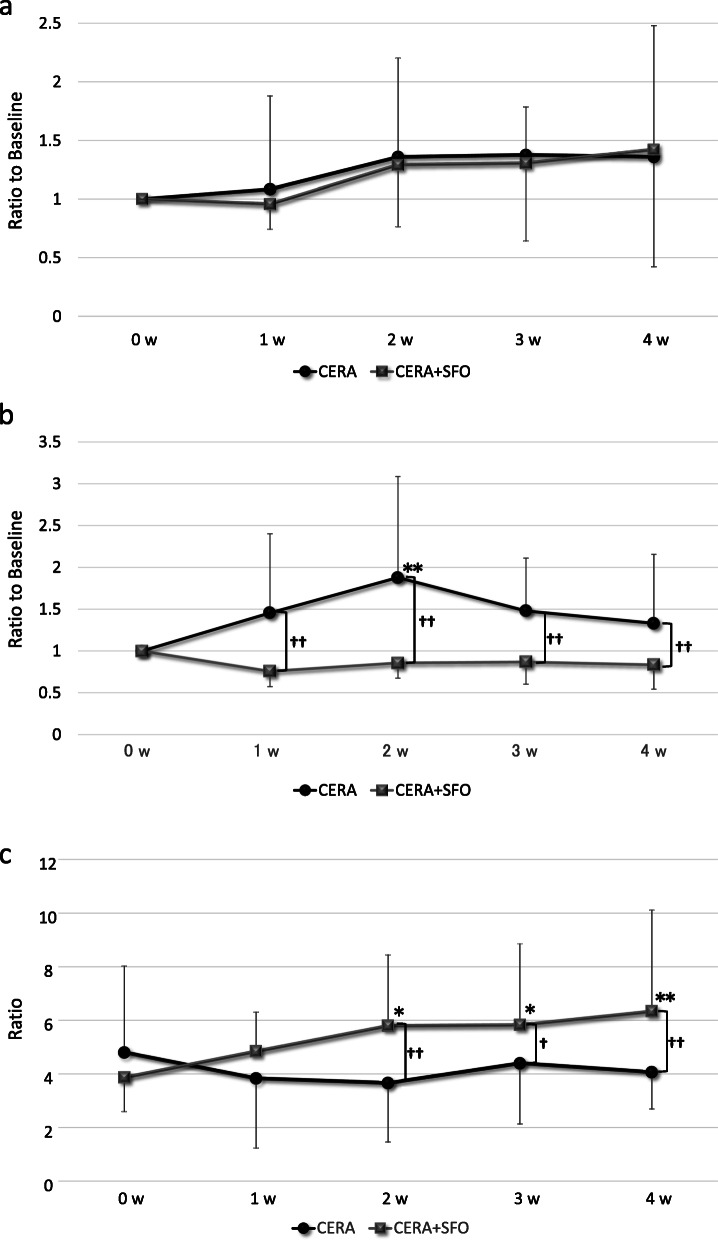
**a**. Temporal changes in iFGF23 compared to baseline. Means ± standard deviations of the ratios to baseline are shown. Abbreviations: iFGF23 intact fibroblast growth factor 23, CERA epoetin beta pegol, SFO saccharated ferric oxide, w week. **b**. Temporal changes in cFGF23 compared to baseline. Means ± standard deviations of the differences from baseline are shown. ^**††**^*p* < 0.01, **p < 0.01 vs. baseline. Abbreviations: cFGF23 C-terminal fibroblast growth factor 23, CERA epoetin beta pegol, SFO saccharated ferric oxide, w week. **c**. Temporal changes in i/cFGF23 ratio. Means ± standard deviations are shown. ^**†**^*p* < 0.05, ^**††**^*p* < 0.01, *p < 0.05 vs. baseline, **p < 0.01 vs. baseline. Abbreviations: i/cFGF23 ratio of intact fibroblast growth factor 23 to C-terminal fibroblast growth factor 23, CERA epoetin beta pegol, SFO saccharated ferric oxide, w week

Figure 2a shows the temporal changes in iFGF23 levels. Significant changes from baseline were not observed after administration of either CERA or CERA + SFO. No significant differences were found in iFGF23 levels between CERA and CERA + SFO. Although a significant increase in cFGF23 level was observed at week 2 after administration of CERA, no significant changes were observed after that of CERA + SFO (Fig. 2b). Administration of CERA alone caused significantly higher levels of cFGF23 than that of CERA + SFO at weeks 1 to 4. Although the i/cFGF23 ratio did not significantly change after administration of CERA, significant increases from baseline were observed at weeks 2 to 4 after administration of CERA + SFO, leading to significantly higher levels of i/cFGF23 ratio than with CERA alone at weeks 2 to 4 (Fig. 2c).

### Changes in inflammatory markers over time

**Fig. 3:**
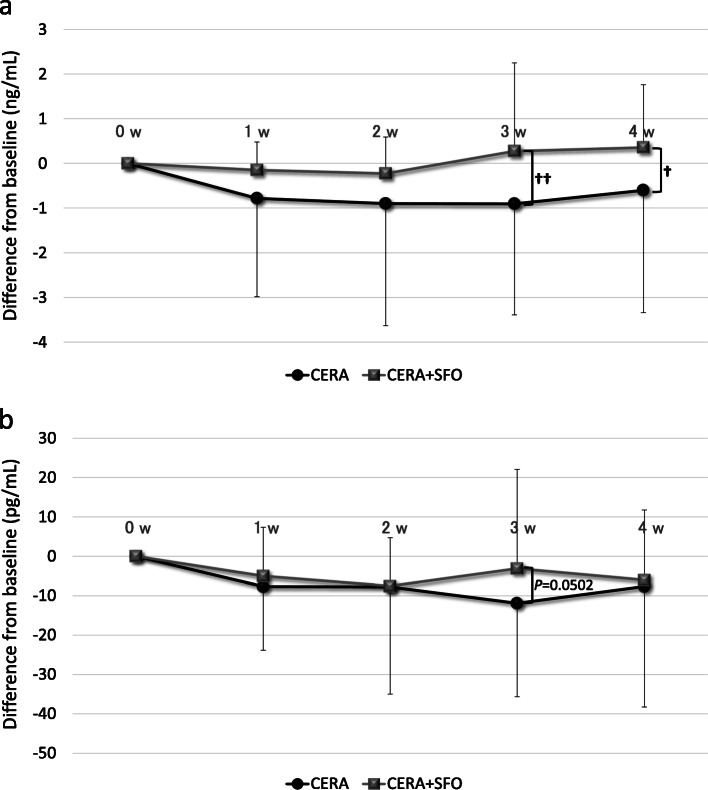
**a**. Temporal changes in hsCRP from baseline. Means ± standard deviations of the differences from baseline are shown. ^**†**^*p* < 0.05, **p < 0.01 vs. baseline. Abbreviations: hsCRP high-sensitivity C-reactive protein, CERA epoetin beta pegol, SFO saccharated ferric oxide, w week. **b** Temporal changes in IL-6 from baseline. Means ± standard deviations of the differences from baseline are shown. Abbreviations: IL-6 interleukin-6, CERA epoetin beta pegol, SFO saccharated ferric oxide, w week

Although no significant changes from baseline were observed in hsCRP levels after administration of CERA or CERA + SFO, significantly higher levels of hsCRP were noted after administration of CERA + SFO than with CERA alone at weeks 3 and 4 (Fig. 3a). Regarding IL-6, there were no changes from baseline after either administration of CERA or CERA + SFO (Fig. 3b). IL-6 levels were higher after administration of CERA + SFO than with CERA alone at week 3 (*p* = 0.0502).

## Discussion

In this study, we demonstrated the surprising results that the co-administration of even a single dose of intravenous SFO attenuated the EPO response 3 weeks after CERA administration. In accordance with the changes in Hb, ERFE and Ret levels were lower with CERA + SFO than with CERA. In addition, the strong correlation between ERFE and Ret, and the strong negative correlation between ERFE and HEPC led to the hypothesis that ESA administration increases erythroblasts, which release ERFE to suppress hepcidin expression in hepatocytes and favour iron acquisition for haemoglobin synthesis [7].

It has already been established that iron is indispensable for erythropoiesis and will accelerate the synthesis of haem and haemoglobin [22].. Erythroblasts require large amounts of iron to facilitate high rates of haem synthesis. The final step in the haem biosynthetic pathway occurs on the inner mitochondrial membrane, where iron is inserted into protoporphyrin IX by ferrochelatase (FECH). Iron is not only a substrate of haem biosynthesis, but is also utilized in the formation of the Fe-S cluster proteins in the mitochondria, which regulate the activity of enzymes associated with haem biosynthesis. These enzymes are aconitase, erythroid delta-aminolevulinate synthase, and FECH [23–25]. Thus, it is reasonable to assume that iron would accelerate erythropoiesis. However, administration of intravenous iron (40 mg of SFO) attenuated the EPO response to CERA.

We recently proposed that excessive iron storage could even hamper erythropoiesis or the differentiation of erythroid in the therapy for renal anaemia [1]. In this review, we hypothesised that two possible mechanisms could affect erythropoiesis, that is, an increase in hepcidin levels and the bone marrow environment with oxidative stress.

Iron administration inevitably increases hepcidin levels, which hampers iron recycling from senescent erythrocytes and diminishes the iron supply to erythroblasts sequentially. The role of hepcidin-mediated iron restriction in the development of renal anaemia has been demonstrated in several recent animal studies. In these observations, mice with adenine-induced CKD did not show anaemia or iron deficiency if hepcidin expression was deleted [4, 26]. In the present study, hepcidin levels were significantly higher with CERA + SFO administration than with CERA alone throughout the study period. Therefore, it is reasonable to suspect that the higher hepcidin level was related to the effect of co-administration of iron on erythropoiesis.

Second, a large body of in vitro and in vivo evidence shows that bone marrow iron overload inhibits erythroid differentiation. However, this mechanism may not affect the results of the present study, because decreases in serum ferritin and TSAT after CERA administration may show a decline in the iron storage of bone marrow.

In addition, we should take into account FGF23 metabolism, above all else, from recent observations. Although FGF23 has been established as a phosphaturic hormone, it has been demonstrated to have a negative effect on erythropoiesis, as FGF23 knockout mice showed increased erythropoiesis with reduced erythroid cell apoptosis. FGF23 is formed as an intact, biologically active protein (iFGF23) and proteolytically cleaved into cFGF23 [27]. Cleavage of iFGF23 is regulated by several factors, including EPO, iron, CKD, and inflammation [15, 16]. Although cFGF23 is believed to be inactive, recent animal studies showed that cFGF23 even reduces the suppressive effect of iFGF23 on erythropoiesis by competitively inhibiting FGF23 receptor signalling [17, 18, 28]. In the present study, cFGF23 level was significantly higher in the CERA period than in the CERA+SFO period, whereas iFGF23 level did not differ between CERA and CERA + SFO periods. From these observations, we can speculate that the difference in cFGF23 level between the CERA and CERA+SFO periods may affect erythropoiesis. A single administration of intravenous iron may affect FGF23 cleavage, and consequently, lower cFGF23 levels.

In this study, we also examined the effects of CERA and SFO on inflammatory markers, such as hsCRP and IL-6. Interestingly, a single administration of intravenous iron increased serum levels of hsCRP, but not IL-6. ESA has been reported to have the potential to ameliorate uremic inflammation [29, 30]. Conversely, IL-6 and tumour necrosis factor-alpha levels reportedly increase temporarily after the administration of intravenous iron preparations [31]. Iron is taken up by monocytes, resulting in the activation of the transcription factor, nuclear factor-kappa B, which plays a central role in the immune response and increases inflammatory cytokines [31]. In this study, hsCRP and IL-6 levels did not significantly change after administration of CERA. However, they became higher after administration of CERA + SFO compared with administration of CERA alone. Although the anti-inflammatory effects of CERA were not documented, the inflammatory effects of SFO were observed. The inflammation induced by SFO could, at least in part, account for the attenuation of haematopoiesis.

### Limitations

First, the sample size of this study was small, which could have influenced the results. Second, a single-arm study design is also a major limitation. Third, the measurement of each biomarker is limited to patients undergoing haemodialysis after the administration of CERA and CERA + SFO. The effects of other ESA preparations or oral iron preparations are unknown. Similarly, the effects of patients with CKD not undergoing haemodialysis are also unknown.

## Conclusions

In this study, we examined the effects of co-administration of intravenous iron on erythropoiesis, iron metabolism, and FGF23 metabolism in patients on haemodialysis receiving ESA. Unexpectedly, a single dose of intravenous iron attenuated the EPO response, with the elevation of hepcidin level, i/cFGF23 ratio, and hsCRP level. We presume that iron administration may inhibit erythropoiesis via an increase in hepcidin synthesis, inhibition of FGF23 cleavage, and inflammation. Although the PIVOTAL trial [32] encourages many nephrologists to proactively administer iron, our results would notify them that iron might have opposite effects on hematopoiesis. Further investigation is needed in which iron dosage, routes of iron administration, and iron preparation would be changed and its effects on erythropoiesis examined.

## Supplementary Information

**Additional file 1: Supplementary Figure S1.** Correlation between ERFE and Rec. When all measurements were included, Ln (ERFE) and Ln (Ret) showed a significant positive correlation with r = 0.765 (*p* < 0.0001). Abbreviations: ERFE erythroferrone, Ret reticulocyte. **Supplementary Figure S2.** Correlation between ERFE and HEPC. When all measurements were included, Ln (ERFE) and Ln (HEPC) showed a significant negative correlation with r = − 0.866 (p < 0.0001). Abbreviations: ERFE erythroferrone, HEPC hepcidin-25.

## Data Availability

The datasets used and analyzed during the current study area available from the corresponding author on reasonable request.

## Abbreviations

Alb: Albumin
CCa: Corrected calcium
CERA: Epoetin beta pegol
cFGF23: C-terminal fibroblast growth factor 23
CHr: Reticulocyte haemoglobin content
CKD: Chronic kidney disease
ELISAs: Enzyme-linked immunosorbent assays
EPO: Erythropoietin
ERFE: Erythroferrone
ESAs: Erythropoiesis-stimulating agents
FECH: Ferrochelatase
FGF23: Fibroblast growth factor 23
FRN: Ferritin
Hb: Haemoglobin
HEPC: Hepcidin-25
hsCRP: High-sensitivity C-reactive protein
i/cFGF23 ratio: Ratio of intact fibroblast growth factor to C–terminal fibroblast growth factor 23
iFGF23: Intact fibroblast growth factor 23
IL-6: Interleukin 6
IP: Inorganic phosphorus
LC/MS/MS: Liquid chromatography-tandem mass spectrometry
Ret: Reticulocytes
SFO: Saccharated ferric oxide
TSAT: Transferrin saturation, Hb, iFGF23, cFGF23
Whole PTH: Parathyroid hormone 1–84
