# Supplementary material for: Saccharated ferric oxide attenuates haematopoietic response induced by epoetin beta pegol in patients undergoing haemodialysis
Source: BMC Nephrol. 2021 Apr 8;22:124. doi: 10.1186/s12882-021-02320-2 (PMC8034147; doi:10.1186/s12882-021-02320-2)
Supplement: Supplementary file 1 — Additional file 1: Supplementary Figure S1. Correlation between ERFE and Rec. When all measurements were included, Ln (ERFE) and Ln (Ret) showed a significant positive correlation with r = 0.765 (p < 0.0001). Abbreviations: ERFE erythroferrone, Ret reticulocyte. Supplementary Figure S2. Correlation between ERFE and HEPC. When all measurements were included, Ln (ERFE) and Ln (HEPC) showed a significant negative correlation with r = − 0.866 (p < 0.0001). Abbreviations: ERFE erythroferrone, HEPC hepcidin-25. [file 12882_2021_2320_MOESM1_ESM.pptx]

## Slide 1
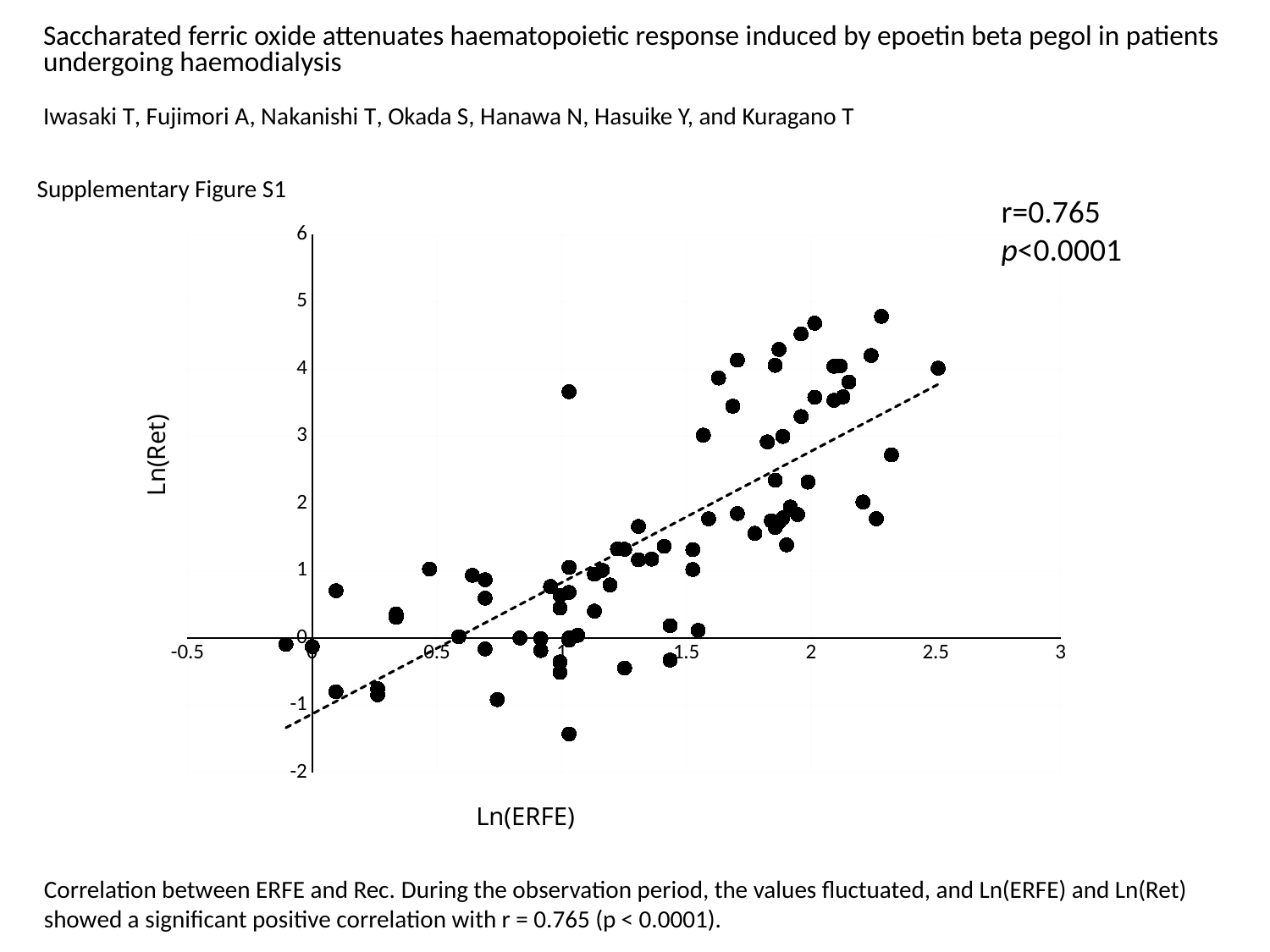

Saccharated ferric oxide attenuates haematopoietic response induced by epoetin beta pegol in patients
undergoing haemodialysis
Iwasaki T, Fujimori A, Nakanishi T, Okada S, Hanawa N, Hasuike Y, and Kuragano T
Supplementary Figure S1
r=0.765
p<0.0001
### Chart
| Category | Ln(ERFE) |
|---|---|Ln(Ret)
Ln(ERFE)
Correlation between ERFE and Rec. During the observation period, the values fluctuated, and Ln(ERFE) and Ln(Ret)
showed a significant positive correlation with r = 0.765 (p < 0.0001).

## Slide 2
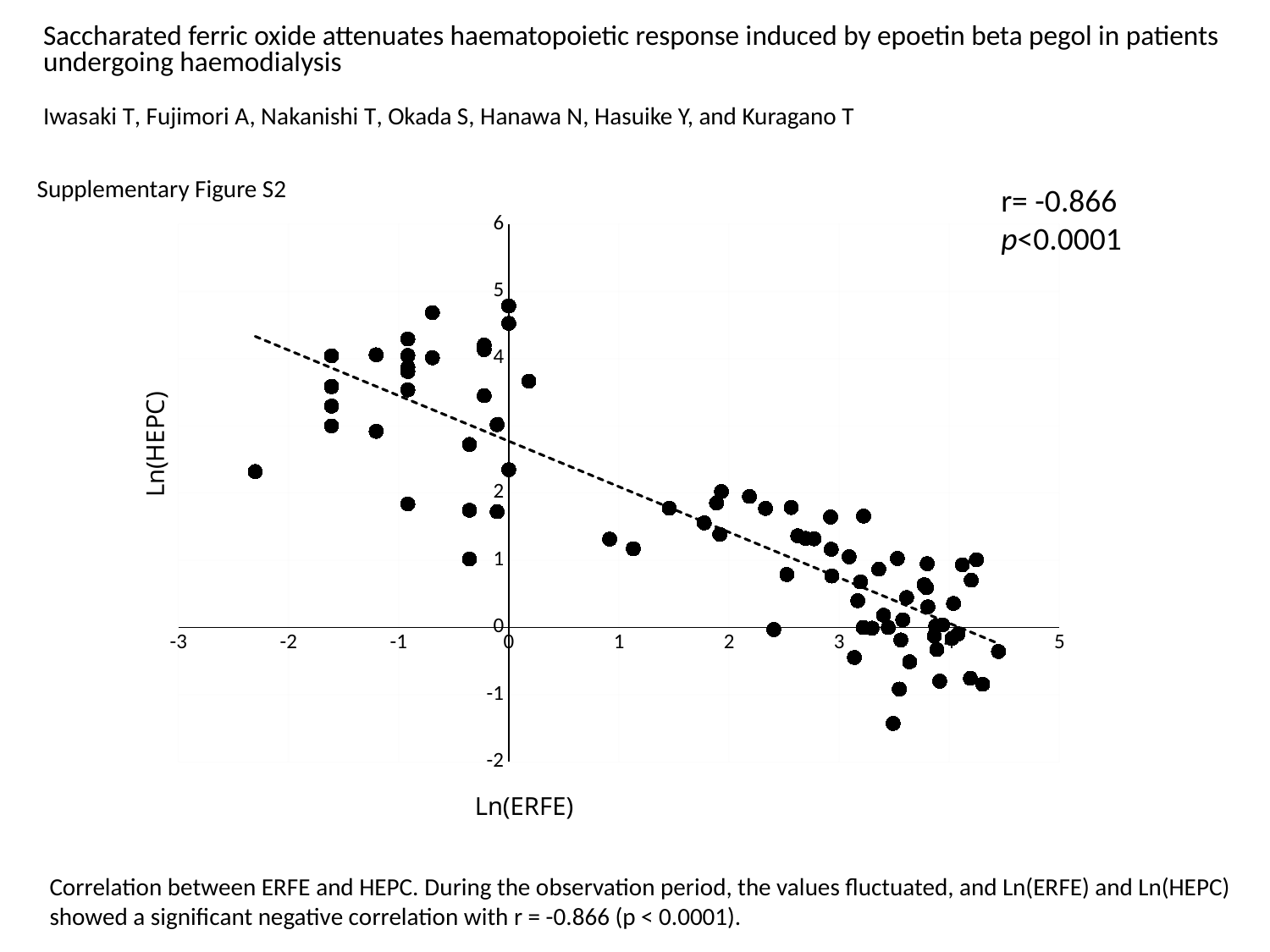

Saccharated ferric oxide attenuates haematopoietic response induced by epoetin beta pegol in patients
undergoing haemodialysis
Iwasaki T, Fujimori A, Nakanishi T, Okada S, Hanawa N, Hasuike Y, and Kuragano T
Supplementary Figure S2
r= -0.866
p<0.0001
### Chart
| Category | Ln(ERFE) |
|---|---|Ln(HEPC)
Ln(ERFE)
Correlation between ERFE and HEPC. During the observation period, the values fluctuated, and Ln(ERFE) and Ln(HEPC)
showed a significant negative correlation with r = -0.866 (p < 0.0001).
